# Clinical outcomes and quality of life assessment of fully laparoscopic appendiceal flap and tongue mucosa ureteroplasty for complex ureteral strictures

**DOI:** 10.3389/fsurg.2026.1761830

**Published:** 2026-03-10

**Authors:** Yuli Luo, Hongzhi Fang, Changjian Shi, Jie Xu, Xinyi Li, Yunfei Li

**Affiliations:** Department of Urology, Renmin Hospital, Hubei University of Medicine, Shiyan, Hubei, China

**Keywords:** appendiceal flap, lingual mucosal graft, quality of life, ureteral stricture, ureteroplasty

## Abstract

**Purpose:**

The main goal of this study is to evaluate the clinical effectiveness of two fully laparoscopic methods. These two methods are appendiceal flap ureteroplasty (AFU) and lingual mucosal graft ureteroplasty (LMGU). They are used to treat complex ureteral strictures (US). This study also analyzes how these methods affect the health-related quality of life (HRQoL) of patients.

**Methods:**

We did a single-center, retrospective cohort study. This study included 22 patients who had complex US. All these patients received fully laparoscopic AFU or LMGU in our hospital. The time of the surgery was from January 2022 to October 2024. We assessed surgical results. We based the assessment on radiographic imaging, renal function tests and patient-reported outcomes. Patient-reported outcomes were longitudinally evaluated using the internationally validated 36-Item Short Form Health Survey (SF-36) at one day before surgery, 6 months, and 12 months postoperatively.

**Results:**

All 22 patients successfully underwent the fully laparoscopic procedures without conversion to open surgery. The cohort comprised 14 patients who received AFU and 8 who received LMGU. The average length of US in the patients was 4.14 ± 0.68 cm. The average time spent on surgery was 198.86 ± 44.88 min. The median estimated blood loss during surgery was 67.5 ml. The median number of days patients stayed in the hospital after surgery was 8 days. The median follow-up period for the patients was 12 months. Every surgery was successful in terms of technique, so the success rate reached 100%. Patient-reported outcome scores showed obvious improvement. This improvement happened from the baseline to 6 months after surgery, and it also happened at the 12-month postoperative evaluation. Most domains of the scores had this improvement, and the difference was statistically significant (*p* < 0.05).

**Conclusions:**

Both fully laparoscopic AFU and LMGU are safe and effective for the reconstructive treatment of complex US. We also found that HRQoL improved significantly after the operation.

## 1 Introduction

Ureteral strictures are considered a common organic lesion in urological practice and have been linked to diverse etiologies, including congenital anomalies, iatrogenic injury, trauma, inflammatory sequelae of stone disease, and chronic infection (1). If left untreated, impaired urinary drainage can result in hydronephrosis, potentially leading to progressive renal impairment. In advanced stages, this may progress to irreversible renal failure, which is frequently complicated by recurrent urinary tract infections and flank or abdominal pain (2).These sequelae significantly impair HRQoL. Among US, the complex US has long represented a major therapeutic challenge in urology due to its intricate anatomy and the technical difficulties associated with its repair (3). Complex US is defined as ureteral luminal narrowing in which the stricture segment is sufficiently long (≥2 cm) to preclude direct end-to-end anastomosis, or in which severe tissue damage and scar adhesions complicate intraoperative localization of the lesion, which consequently increases surgical difficulty. At present, there is no consistent standard for managing complex US; treatment selection in clinical practice requires comprehensive consideration of stricture characteristics (location and length), renal function status, and surgeon expertise. Historically recognized as the gold standard for ureteral reconstruction, traditional open ureteroplasty is nonetheless associated with significant limitations, such as considerable surgical trauma, significant blood loss, prolonged postoperative recovery periods, and an increased risk of incision-related complications (4). Endoscopic incision and balloon dilation are often the preferred initial minimally invasive treatments for US. For complex strictures that are not amenable to endoscopic management, laparoscopic and robot-assisted ureteroplasty have been established as mainstream reconstructive options, which offer the benefits of reduced invasiveness and faster postoperative recovery (5). The Boari bladder flap procedure is primarily utilized for lower US; however, it fails to provide sufficient reach for the reconstruction of the proximal ureter (6). The native ureter is considered the optimal material for repair. Consequently, end-to-end anastomosis represents the preferred reconstructive approach for short-segment strictures located in the proximal or mid-ureteral regions (7). However, these approaches exhibit limited efficacy for long-segment strictures and are generally contraindicated in complex cases requiring extensive resection of scar tissue (8). When long-segment upper US are not amenable to repair via direct anastomosis, reconstruction typically requires ileal ureter replacement or renal autotransplantation. However, both procedures are highly complex and are associated with significant risks, including complications related to the use of bowel segments and the challenges of vascular anastomosis (9, 10). Ureteroplasty is a well-established surgical procedure that is designed to relieve ureteral obstruction, restore urinary tract function, alleviate symptoms, and preserve renal function. Recently, the application of appendiceal flaps and lingual mucosal grafts has gained increasing interest as innovative and effective techniques for reconstructing complex US (11–15). Additionally, buccal mucosa graft (BMG) ureteroplasty is a safe and effective surgical approach for treating complex ureteral strictures (16, 17). The appendiceal flap (AF), recognized for its robust vascularity, preservation of intestinal continuity, and association with rapid postoperative recovery, has been successfully employed in urethral reconstruction (18). The lingual mucosal graft (LMG) has the advantages of easy access, simple harvesting, and strong resistance to infection. Owing to its favorable immunological profile and excellent tissue compatibility, it serves as an ideal reconstructive material, facilitating rapid graft integration and revascularization. Consequently, LMGs have been extensively utilized in urethral reconstruction, where they have demonstrated favorable functional outcomes (19).

Nevertheless, robust clinical studies evaluating the application of these two grafts in completely laparoscopic reconstruction for complex US remain scarce. Current literature is primarily focused on surgical safety and short-term patency rates. Moreover, outcome assessments predominantly rely on objective clinical metrics, with comparatively less emphasis on patient-reported health-related quality of life (HRQoL). A systematic assessment of HRQoL both preoperatively and postoperatively provides a crucial patient-centered perspective for the comprehensive evaluation of surgical efficacy. A comprehensive HRQoL assessment encompasses multiple domains, including physical function, emotional and psychological well-being, and social functioning (20). Longitudinal assessment of HRQoL offers valuable insights into the treatment outcomes for patients undergoing ureteroplasty. Nevertheless, robust evidence in this specific domain remains scarce.

We conducted a single-center retrospective study. We analyzed the clinical data of patients who presented with complex US and underwent fully laparoscopic AFU or fully laparoscopic LMGU between January 2022 and October 2024. The present study was designed to assess the objective surgical efficacy and safety of these fully laparoscopic approaches, as well as to perform a longitudinal evaluation of HRQoL outcomes focusing on the preoperative and postoperative periods.

## 2 Materials and methods

### 2.1 Study population

We enrolled 22 patients in this retrospective study. These patients were diagnosed with complex US. They had fully laparoscopic AFU or LMGU at our institution. between January 2022 and October 2024. The indications for surgical intervention were persistent symptoms, imaging-confirmed obstruction with functional impairment, and/or deteriorating renal function. We set the inclusion criteria of this study like this: (1) Patients had complex US. (2) preoperative confirmation of stricture characteristics and hydronephrosis severity by computed tomography (CT) or urography; (3) availability of complete clinical and follow-up data; and (4) provision of informed consent. Patients were excluded for any of the following: (1) concurrent malignancy, severe cardiopulmonary/hepatic/renal dysfunction, or coagulation disorders; (2) uncontrolled active urinary tract infection; (3) history of renal autotransplantation or ileal ureter reconstruction, which could confound outcomes; or (4) incomplete follow-up data. The study protocol got approval from the Institutional Review Board of Shiyan People's Hospital. Written informed consent was acquired from every individual participant.

### 2.2 Data collection

Data on demographic characteristics, perioperative factors, and follow-up outcomes were collected. The laterality and etiologies of US were determined through a comprehensive review of clinical histories and imaging findings. Surgical success was defined as the concurrent presence of two criteria: First, the patient had no related symptoms. Second, there was no radiographic evidence of obstruction. We used the Medical Outcomes Study SF-36 to evaluate HRQoL. Patients filled out the SF-36 by themselves. They did this one day before surgery. They also did it at 6 months and 12 months after the operation.

### 2.3 Surgical techniques

#### 2.3.1 Preoperative preparation

Initially, all enrolled patients underwent percutaneous nephrostomy and removal of any preexisting double-J stents, with the aim of alleviating hydronephrosis and decompressing the obstructed urinary system. We waited for two weeks. This time let ureteral edema go away. Then we did antegrade and retrograde urography. These tests helped us clearly find the anatomical location and length of the ureteral stricture. We routinely did radionuclide renal scintigraphy. This test helped us measure the split renal function of the affected kidney. Computed tomography urography (CTU) or magnetic resonance urography (MRU) was utilized to characterize the periductal conditions and identify imaging features suggestive of malignancy. All patients underwent ureteroscopy for direct macroscopic assessment of the stricture site. For cases in which malignancy could not be definitively excluded by imaging or endoscopy, urinary cytology and/or targeted biopsy of suspicious lesions was performed to rule out malignancy. Patients scheduled for LMGU initiated preoperative oral preparation with chlorhexidine mouthwash 1–2 days before surgery to reduce the bacterial load. A preoperative cleansing enema was administered on the day before surgery. Standard prophylactic intravenous antibiotics were administered intraoperatively.

#### 2.3.2 Patient positioning and trocar placement

All surgical procedures were carried out under general anesthesia with endotracheal intubation. A urinary catheter was placed and retained. Then, 50 mL of diluted methylene blue was instilled into the bladder, and the catheter was clamped. The patient was placed in a lateral decubitus position at an angle of 45°–60°, with the contralateral (unaffected) side positioned inferiorly. We created pneumoperitoneum first. Then we put a 10 mm trocar into the umbilical margin. This trocar was used to hold the 30° laparoscope. We placed two more 12 mm trocars under direct visualization. One trocar went at the midclavicular line. It was about 3 cm below the umbilicus. The other trocar was 5 cm below the costal margin. It was along the lateral edge of the rectus abdominis muscle.

#### 2.3.3 Free-hanging narrow segment of the ureter

**Figure 1:**
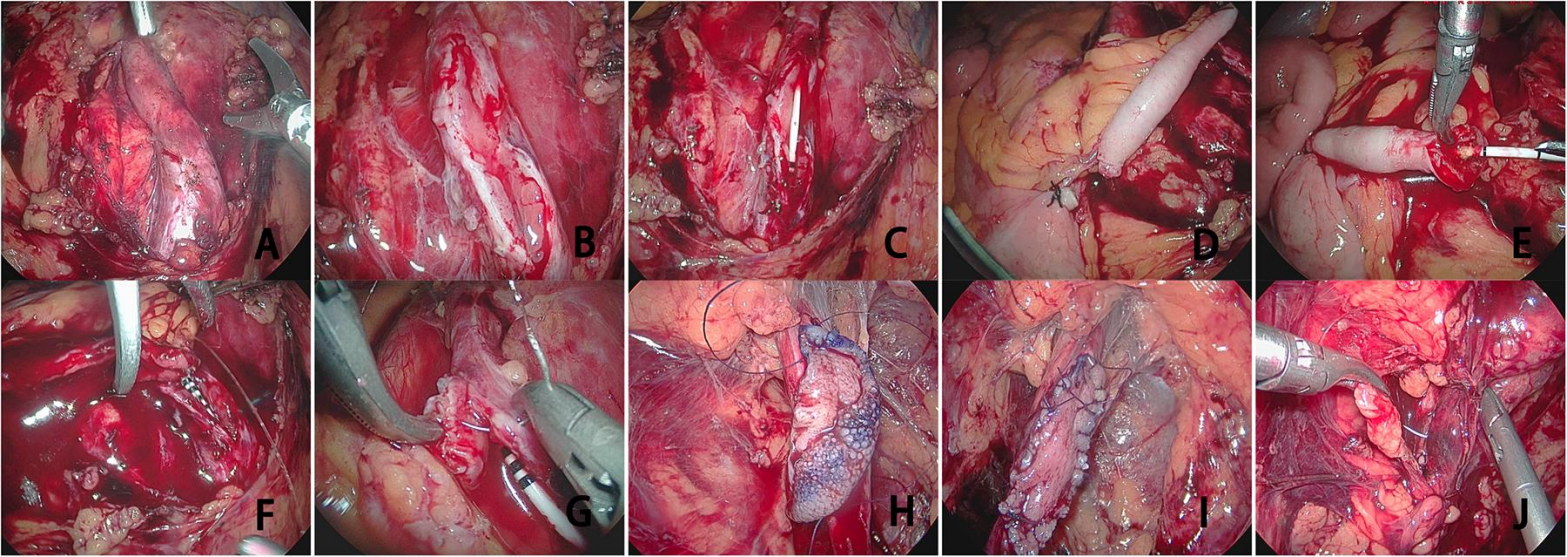
Brief surgical steps for lingual mucosa ureteroplasty and appendiceal flap ureteroplasty. **(A)** Thoroughly dissect and expose the narrowed segment of the ureter. **(B)** Make a longitudinal incision through the lumen of the narrowed ureter to the normal mucosa. **(C)** Measure the length of the narrowed segment and place a double-J stent. **(D)** Dissect the appendiceal mesentery and transect the appendix. **(E)** Incise the appendix along the opposite mesenteric margin to create a patch graft. **(F)** Suture the two ends of the appendix to the corresponding notches on both ends of the ureter. **(G)** Continuously suture both sides of the appendiceal flap to the narrowed ureteral wall. **(H)** Suture and secure the lingual mucosa to the proximal end of the narrowed ureter. **(I)** Perform interrupted/continuous anastomosis between the ureter and lingual mucosa. **(J)** Enclose the anastomotic site with surrounding adipose tissue or omentum. LMG, lingual mucosal graft, AF, appendiceal flap.

The lateral peritoneal layer was incised along the white line of Toldt within the paracolic gutter. The transverse colon was then mobilized by dissecting along its superior margin and dividing the hepaticocolic ligament. The colon was retracted medially and inferiorly to expose the retrorenal space and the anterior lamina of the renal fascia.The anterior lamina of renal fascia was incised to identify the dilated, hydronephrotic proximal ureteral segment. The diseased ureter was then meticulously dissected to fully expose the narrowed segment (Figure 1A). A ventral longitudinal incision was made through the stricture until normal-caliber ureteral lumen and healthy mucosa were encountered (Figure 1B). Measure the length of the narrowed segment of the ureter. We then placed a double-J ureteral stent. We checked two things. First, the proximal end of the stent was in the renal pelvis. Second, the distal end was in the urinary bladder (Figure 1C). The aforementioned steps for identifying and dissecting the ureteral stricture segment were identical for both the AF and LMG procedures.

#### 2.3.4 Acquire the graft patches

Preparation of the Appendiceal Flap Patch: The appendix was assessed intraoperatively for gross morphology, vascular integrity, and length adequacy to confirm its suitability for reconstruction. The appendix was carefully transected at its base from the cecum, with meticulous preservation of the mesoappendix and vascular pedicle. The mesentery along the ascending colon was then partially mobilized to achieve sufficient mobility and tension-free reach of the appendiceal flap (Figure 1D). We then cut the appendix lengthwise. We did this along its antimesenteric border. This step made a flat mucosal patch with good blood supply (Figure 1E). The luminal surface of the appendiceal patch was gently cleaned with povidone-iodine-soaked gauze. The patch was then trimmed to match the length of the incised ureter.

Harvesting of the Lingual Mucosal Graft: With the patient's head turned toward the affected side, the facial and oral regions were disinfected with povidone-iodine. A mouth gag was placed to gently open the oral cavity, and the tongue was retracted to expose the donor site. The planned graft area on the ventral and lateral tongue surface was outlined with methylene blue. Inject the epinephrine-containing lidocaine solution into the submucosal layer. The marked mucosa was then sharply excised using a scalpel blade. We closed the donor site defect with a running suture. We used 3-0 absorbable material for this. The harvested graft was meticulously trimmed of any residual submucosal or muscular tissue and stored in sterile normal saline until transplantation.

#### 2.3.5 Graft ureteroplasty

##### 2.3.5.1 AFU

The ureteral defect was covered with the prepared appendiceal flap, orienting it such that the serosal surface faced the lumen. We first fixed the proximal and distal ends of the mucosal flap. We used interrupted 5-0 polydioxanone sutures for this. The flap ends were fixed to the matching apices of the incised ureter (Figure 1F). Subsequently, the lateral margins of the flap were approximated to the edges of the ureteral defect with a running suture of the same material, completing the Inlay repair (Figure 1G).

##### 2.3.5.2 LMGU

We placed the lingual mucosal graft. Its mucosal surface faced the ureteral lumen. The proximal and distal extremities of the harvested lingual mucosal graft were initially secured to the corresponding apices of the incised ureter using interrupted 5-0 polydioxanone sutures (Figure 1H). We then connected the medial and lateral edges of the graft to the corresponding margins of the ureteral defect. We used a running 5-0 polydioxanone suture for this (Figure 1I). After completing the anastomosis, a leak test was performed by injecting saline through the pre-placed nephrostomy tube. Intravenous furosemide could be administered to augment diuresis and challenge the repair under higher pressure. Any observed leakage was repaired with additional interrupted sutures. We finally wrapped the reconstructed ureteral segment. We used a pedicled omental flap or perirenal adipose tissue for this. This step gave extra vascular support and isolated the anastomosis (Figure 1J).

### 2.4 Quality of life scale

SF-36 Health Survey (Chinese Version 2.0): We evaluated HRQoL using the SF-36 (21). This is a validated generic patient-reported outcome measure. The SF-36 has 36 items. These items are grouped into eight separate domains. Their standardized abbreviations are as follows: Physical Functioning (PF), Role-Physical (RP), Bodily Pain (BP), General Health (GH), Vitality (VT), Social Functioning (SF), Role-Emotional (RE), and Mental Health (MH), Each domain generates a raw score that is linearly transformed to a scale of 0–100, where higher scores indicate better HRQoL in the corresponding domain. Each domain is represented by 2–10 items. We linearly transformed each domain score to a standardized 0–100 scale. Higher scores on this scale mean better HRQoL outcomes.

### 2.5 Statistical analysis

We used the Shapiro–Wilk test to check if continuous variables followed a normal distribution. Normally distributed continuous data are reported as mean ± standard deviation (SD). Non-normally distributed continuous variables are summarized as median [interquartile range (IQR); full range]. Categorical variables are described using frequencies (percentages). The HRQoL scores of the study cohort were compared with established normative data from the general Chinese population using an independent samples *t*-test (22). The Friedman test was employed to analyze longitudinal changes in HRQoL scores across three time points: preoperative, 6-month postoperative, and 12-month postoperative. The Wilcoxon signed-rank test was utilized for pairwise comparisons, specifically to compare postoperative scores at each follow-up interval with preoperative baseline values. A paired t-test was applied to evaluate differences between preoperative and postoperative serum creatinine levels. Statistical significance was defined as a two-tailed *P*-value < 0.05. All statistical analyses were performed using SPSS Statistics 27.0 and R 4.3.0 software.

## 3 Results

As shown in Table 1, our study included 22 patients. There were 14 males (63.64%) and 8 females (36.36%), with a mean age of 60.05 ± 9.75 years. Left-sided US were identified in 6 patients (27.27%), while right-sided strictures were observed in 16 patients (72.73%). The stricture location was proximal in 15 (68.18%) patients, mid-ureteral in 6 (27.27%), and distal in 1 (4.55%). Etiologies included ureteral calculi in 11 patients (50.00%), idiopathic causes in 6 patients (27.27%), and iatrogenic injury (resulting from prior abdominal/pelvic surgery) in 5 patients (22.73%). All patients with stone-related strictures had a history of one or more prior endoscopic ureterolithotripsy procedures. The mean length of the ureteral strictures (US) was 4.14 ± 0.68 cm (range, 3.00–5.50 cm).

**Table 1:** Patient characteristics.

Characteristic	Overall	AF group	LMG group
Number of patients, *n*	22	14	8
Age, years, Mean ± SD	60.05 ± 9.75	61.21 ± 10.59	58.00 ± 8.33
Gender, *n* (%)			
Male	14 (63.64)	7 (50.00)	7 (87.50)
Female	8 (36.36)	7 (50.00)	1 (12.50)
BMI, kg/mm^2^, Mean ± SD	24.98 ± 2.85	24.66 ± 2.39	25.51 ± 3.59
Laterality, *n* (%)			
Left	6 (27.27)	0 (0.00)	6 (75.00)
Right	16 (72.73)	14 (100.00)	2 (25.00)
Stricture Location, *n* (%)			
Proximal	15 (68.18)	10 (71.43)	5 (62.50)
Middle	6 (27.27)	4 (28.57)	2 (25.00)
Distal	1 (4.55)	1 (12.50)	0 (0.00)
Presenting symptoms, *n* (%)			
Flank pain	16 (72.73)	10 (71.43)	6 (75.00)
Asymptomatic	6 (27.27)	4 (28.57)	2 (25.00)
Stricture etiology, *n* (%)			
Ureteral stones	11 (50.00)	7 (50.00)	4 (50.00)
Idiopathic stricture	6 (27.27)	3 (21.43)	3 (37.50)
History of endoscopic ureteral surgery, *n* (%)	11 (50.00)	7 (50.00)	4 (50.00)
History of abdominopelvic surgery, *n* (%)	5 (22.73)	4 (28.57)	1 (12.50)
Length, Mean (range)	4.14 (3.00–5.50)	4.29 (3.50–5.50)	4.75 (3.00–8.00)

Fourteen patients underwent AFU, while eight underwent LMGU. Perioperative outcomes are summarized in Table 2. All 22 fully laparoscopic ureteral reconstructive surgeries were successfully completed. No intraoperative complications occurred, and none of the procedures required conversion to open surgery. For the lingual mucosal grafts, the mean dimensions were 5.00 cm (range: 3.50–6.50 cm) in length and 1.50 cm (range: 1.50–2.00 cm) in width. The mean operative time was 198.86 ± 44.88 min (range: 120–300 min), with a median estimated blood loss of 67.50 mL (range: 50–150 mL). The mean postoperative hospital stay was 8.91 ± 2.39 days (range: 7–16 days). No major postoperative complications (Clavien-Dindo classification grade III or higher) were documented during the in-hospital and 30-day postoperative follow-up periods. Three patients developed postoperative fever (Clavien-Dindo grade I), which resolved promptly with antibiotic therapy. One patient in the LMGU group reported transient mild numbness at the tongue donor site, which had completely resolved in all patients by the one-week postoperative follow-up. No patients reported pain, dysarthria, or eating difficulties at that time.

**Table 2:** Perioperative data of patients.

Variables	Overall	AF group	LMG group
Number of patients, *n*	22	14	8
Width of graft (cm), Median (range)	1.50 (1.50–2.00)	1.50 (1.50–1.50)	2.00 (2.00–2.00)
Length of graft, Median (range)	5.00 (3.50–6.50)	5.50 (4.00–6.50)	4.75 (3.50–5.50)
Operative time (min), Mean ± SD	198.86 ± 44.88	179.64 ± 30.54	232.50 ± 47.73
Estimated blood loss (ml), Median (range)	67.50 (50–150)	57.50 (50–100)	77.50 (50–150)
Follow-up time (months), Median (range)	12.00 (12–20)	12.00 (12–18)	12.00 (12–20)
Postoperative hospital stays (days), Mean (range)	8.91 (7–16)	8.50 (7–14)	7.00 (7–16)
Postoperative complications, *n* (%)	4 (18.18)	2 (14.29)	2 (25.00)
Success rate, *n* (%)	22 (100.00)	14 (100.00)	8 (100.00)

**Figure 2:**
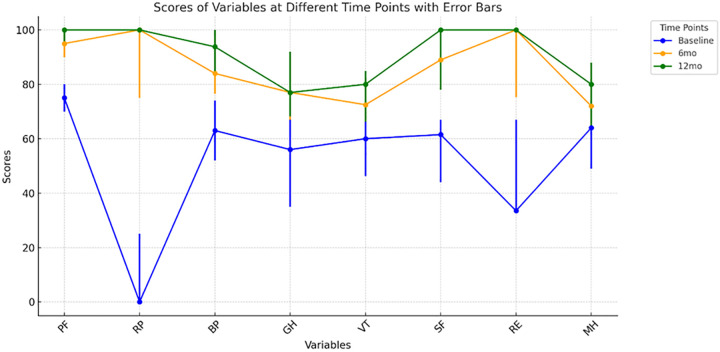
Scores of variables at different time points with error bars. As time progressed, overall improvements were observed across various health-related indicators, with data stability gradually increasing.

The median follow-up duration was 12 months (range: 12–20). In all patients, the ureteral stents were removed cystoscopically on an outpatient basis 6–8 weeks after surgery. Surgical success was defined as the concurrent fulfillment of the following criteria: (a) radiographic improvement on follow-up imaging, (b) patency of the reconstructed ureter, and (c) the absence of symptoms including fever, flank pain, or lumbar discomfort. All 22 patients (100.00%) achieved both clinical and radiographic success at the latest follow-up. Serum creatinine levels were monitored during the postoperative follow-up period. We used a paired t-test to compare serum creatinine levels before and after surgery. A statistically significant reduction was noted: preoperative levels were 99.75 ± 34.50 µmol/L, whereas postoperative levels were 75.87 ± 23.05 µmol/L (*P* < 0.001). No severe complications, including recurrent stricture, ureteral fistula, or graft necrosis, were encountered during the follow-up period. A comparison of preoperative HRQoL scores Comparisons between the study cohort and the general Chinese population are summarized in Table 3. Preoperatively, patients exhibited significantly impaired HRQoL. Scores across all SF-36 domains were notably lower, except for Vitality (VT) and General Health (GH) (all *P* < 0.001). Figure 2 shows the longitudinal changes in HRQoL domain scores. These scores were assessed at three time points: preoperative, 6-month postoperative, and 12-month postoperative. Table 4 presents the longitudinal changes in HRQoL scores across all SF-36 domains. These scores were compared from preoperative baseline to two postoperative follow-up time points: 6 months and 12 months. Comparisons with preoperative baseline demonstrated statistically significant improvements in the median scores of all SF-36 domains at postoperative follow-up (*P* < 0.05). A notable exception was the Mental Health (MH) domain, which did not show a significant improvement at the 6-month assessment but reached statistical significance by the 12-month follow-up. All other domains demonstrated significant improvements at both the 6- and 12-month time points compared to baseline.

**Table 3:** Comparison of preoperative HRQoL scores in patients with complex US (*n* = 22) and Chinese general population (*n* = 3,214).

Dimension	Mean study population ± SD	Mean general population + SD	*p* value (*t* test)
PF	70.45 ± 14.38	94.02 ± 12.44	<0.001
RP	22.73 ± 20.28	88.79 ± 28.49	<0.001
BP	61.73 ± 16.40	88.18 ± 19.02	<0.001
GH	55.18 ± 19.28	69.74 ± 20.95	0.001
VT	55.68 ± 13.91	68.92 ± 18.78	0.001
SF	56.14 ± 18.22	88.03 ± 16.00	<0.001
RE	44.00 ± 21.74	89.57 ± 27.95	<0.001
MH	60.00 ± 14.81	77.61 ± 15.85	<0.001

HRQoL, health-related quality of life; US, ureteral stricture; PF, physical function; RP, role-physical; BP, bodily pain; GH, general health; VT, vitality; SF, social functioning; RE, role-emotional; MH, mental health.

**Table 4:** HRQoL scores variation in the different domains during the follow-up [median and (IQR)].

Variables	Baseline	6 months	12 months	*P*
PF	75 (70–80)	95 (90–98.8)^a^	100 (95–100)^b^	<0.001
RP	0 (0–25)	100 (75–100)^a^	100 (100–100)^b^	<0.001
BP	63 (52–74)	84 (76.5–100)^a^	93.8 (84–100)^b^	<0.001
GH	56 (35–75)	77 (67–92)^a^	77 (68.3–92)^b^	0.001
VT	60 (46.3–68.8)	72.5 (66.3–82.5)^a^	80 (66.3–85)^b^	<0.001
SF	61.5 (44–67)	89 (78–100)^a^	100 (78–100)^b^	<0.001
RE	33.5 (33–67)	100 (75.3–100)^a^	100 (100–100)^b^	<0.001
MH	64 (49–72)	72 (64–88)	80 (64–88)^b^	0.009

*p*-value, *p*-value determined by Friedman test comparing score changes during follow-up.

^a^ Indicates a difference between the 6-month postoperative score and baseline value.

^b^ Indicates a difference between the 12-month postoperative score and baseline value. Pairwise comparisons underwent Bonferroni correction.

## 4 Discussion

Complex US, defined as strictures with extensive narrowing, severe anatomical distortion, or a history of repeated treatment failure, have long posed a major challenge in urological reconstruction. Although open ureteroplasty has historically achieved favorable outcomes, its considerable invasiveness, prolonged recovery, and higher risk of incision-related complications have led to its gradual replacement by minimally invasive approaches. Endoscopic management, including balloon dilation and endoscopic incision, remains the first-line minimally invasive option; however, its efficacy is limited in long-segment strictures (>2 cm) or recurrent cases, with reported recurrence rates ranging from 30% to 50% (5). Reconstructive techniques such as the Boari bladder flap and end-to-end ureteral anastomosis are often constrained by stricture location and length, limiting their applicability in complex repair scenarios (7). Nevertheless, both ileal ureter replacement and renal autotransplantation have restricted clinical utility due to substantial technical complexity and the potential for serious complications (23). Commonly used autologous materials for ureteral reconstruction include oral mucosal grafts (obtained from the lingual or buccal region) and appendiceal flaps. Buccal mucosa shares similar characteristics with lingual mucosa, both demonstrating excellent elasticity and a robust vascular network. Postoperative metabolic complications are rare, and multiple institutions have reported successful clinical applications of both graft materials (16, 24). The appendiceal flap and lingual mucosal graft ureteroplasty techniques evaluated in this study offer viable alternatives that overcome several limitations of conventional approaches. Each graft offers unique advantages: the appendiceal flap demonstrates minimal electrolyte absorption and controlled mucus secretion, substantially reducing the risks of metabolic disturbances and mucus-related complications (25). Furthermore, the appendiceal flap preserves its native vascular pedicle, which provides a theoretical advantage by lowering the risk of ischemic necrosis. The luminal diameter and anatomical course of the appendix closely match those of the right ureter, enabling physiological reconstruction. However, the relatively short length of the appendix typically limits its use to right-sided ureteral reconstruction. Notably, several case reports have described successful left-sided ureteral reconstruction using a fully mobilized appendiceal flap with its mesentery (26). The appendiceal flap is not considered viable in patients with acute/chronic appendicitis, inadequate appendiceal dimensions, previous appendectomy, or other contraindications. Lingual mucosal grafts are regarded as an ideal reconstructive material owing to their accessibility, reliable availability, favorable immunological profile, and excellent tissue compatibility. Histologically, lingual mucosa exhibits a thick epithelium, abundant elastic fibers, a thin lamina propria, and robust vascularization—key characteristics that facilitate successful graft integration. Furthermore, as it is naturally adapted to a humid environment, lingual mucosa demonstrates inherent resistance to infection, and the donor site heals favorably with minimal donor-site morbidity (27). In recent years, laparoscopic ureteroplasty has gained widespread clinical acceptance worldwide as a standard surgical approach for the management of complex ureteral strictures. With the increasing adoption of surgical robotic systems, robot-assisted ureteroplasty has emerged as a prominent research focus. Collectively, these techniques constitute the core technological foundation of contemporary minimally invasive ureteral reconstruction. Capitalizing on the minimally invasive benefits of fully laparoscopic techniques, the present study investigates laparoscopic AFU and LMGU. All procedures in this series were successfully performed without conversion to open surgery. The median estimated blood loss was 67.5 mL, and the mean postoperative hospital stay was 8.91 ± 2.39 days. Compared with traditional open ureteroplasty, the fully laparoscopic approach markedly reduced surgical trauma and accelerated recovery, consistent with contemporary principles of minimally invasive and precision surgery.

Perioperative outcomes are key metrics for evaluating surgical safety. In the present series, the mean operative time for the combined procedures was 198.86 ± 44.88 min, comparable to published operative times for laparoscopic ureteroplasty (13, 14). No major intraoperative adverse events were encountered during these technically challenging reconstructive procedures, which included laparoscopic ureteral mobilization, graft harvesting, and anastomosis. Postoperatively, three patients developed low-grade fever (Clavien-Dindo grade I) that resolved promptly with antibiotic therapy. One patient in the lingual mucosal graft group reported transient hypesthesia at the donor site, which resolved completely within one week. No severe complications, including oral dysfunction or graft necrosis, were observed, supporting the safety profile of both graft types in clinical practice. The primary therapeutic goals were relief of obstruction and preservation of renal function. At a median follow-up of 12 months, all enrolled patients achieved the predefined clinical success criteria, resulting in a 100% clinical success rate. Serum creatinine levels decreased significantly from preoperative (99.75 ± 34.50 µmol/L) to postoperative values (75.87 ± 23.05 µmol/L; *p* < 0.001), indicating effective relief of urinary obstruction, prevention of further renal impairment, and partial recovery of renal function. These findings align with prior small-series studies examining appendiceal flap and lingual mucosal graft ureteroplasty for ureteral stricture reconstruction (28).

The primary goal of any surgical intervention is to improve HRQoL. Traditional assessment of ureteral stricture treatment outcomes has overemphasized radiographic patency and objective renal function parameters, with insufficient attention to patient-reported outcomes and quality of life measures. HRQoL is a multidimensional construct that reflects the impact of illness and therapeutic interventions on patients’ physical, psychological, and social well-being. It has emerged as a critical component in comprehensive clinical outcome assessment (20). The present study utilized the internationally validated SF-36 questionnaire to longitudinally assess HRQoL in patients who underwent fully laparoscopic ureteroplasty, with evaluations performed at preoperative baseline, 6 months postoperatively, and 12 months postoperatively. To our knowledge, the present study is among the limited number of investigations in this field that integrate objective therapeutic outcomes with patient-reported outcome measures centered on HRQoL. Preoperatively, patients with complex US had significantly impaired HRQoL compared to the general Chinese population. Scores were lower across all SF-36 domains, except for Vitality (VT) and General Health (GH) (*P* < 0.001). These findings reflect significant quality-of-life impairment attributable to ureteral stricture-related symptoms, including pain, physical functional limitations, and psychological distress—consistent with the typical clinical manifestations of recurrent urinary tract infections, flank pain, abdominal pain, and progressive renal dysfunction (2). During postoperative follow-up, all SF-36 domains—with the exception of Mental Health (MH)—exhibited statistically significant improvements from preoperative baseline at both the 6-month and 12-month assessment time points. The MH domain achieved statistically significant improvement by the 12-month follow-up. This pattern indicates that surgical intervention initially alleviates physical symptoms—including flank pain and dysuria associated with recurrent infections—leading to subsequent improvements in physical and social functioning. Mental health recovery, however, appears to follow a more protracted course, potentially reflecting the time required for psychological adaptation to disease resolution and adjustment to the postoperative state.

Both graft types were associated with substantial HRQoL improvement postoperatively, demonstrating that either appendiceal flap or lingual mucosal graft ureteroplasty can effectively enhance patients’ overall quality of life. These findings extend the clinical value of these reconstructive techniques by demonstrating benefits beyond anatomical and functional restoration to address the fundamental patient-centered outcome of quality of life. This is particularly relevant for complex cases characterized by prolonged symptom duration and multiple prior treatment failures.

Upper urinary tract urothelial carcinoma (UTUC) is a significant malignant factor contributing to ureteral stricture (29). Prior to the clinical application of laparoscopic AFU and LMGU, malignant lesions must be rigorously excluded via systematic evaluations, including imaging studies, endoscopic assessment, and pathological biopsy. Both techniques provide minimally invasive, safe, and effective management for benign complex ureteral strictures, consistent with core oncological safety principles. For rigorously selected patients with primary or recurrent UTUC, these laparoscopic reconstructive techniques effectively restore urinary tract continuity after segmental ureterectomy. They maximize the preservation of affected renal function while ensuring radical tumor resection, thereby demonstrating significant clinical value. However, strict adherence to indications is essential in clinical practice, and intensified long-term postoperative tumor surveillance is required to optimize patient outcomes.

Several limitations should be considered when interpreting our findings. First, the single-center, retrospective study design and limited sample size may compromise the generalizability of the study findings. Second, the subjective nature of questionnaire-based assessments introduces potential for response bias. Third, the relatively short follow-up period necessitates longer-term evaluation to assess the durability of reconstruction and sustainability of quality-of-life improvements. Fourth, the retrospective nature inherently limits control over confounding variables and prevents definitive causal inferences.

Notwithstanding these limitations, the present study provides robust single-center evidence supporting the efficacy of these techniques as valuable treatment options for complex ureteral strictures (US). Future multicenter prospective studies with extended follow-up are warranted to validate long-term outcomes and further guide clinical decision-making for this challenging patient population.

## 5 Conclusions

Fully laparoscopic AFU and LMGU demonstrate favorable safety and feasibility in the reconstructive management of complex ureteral strictures. Both procedures effectively relieve urinary obstruction, preserve renal function, and significantly improve patients’ HRQoL. Given the small sample size, limited follow-up duration, and single-center retrospective design of the present study, these conclusions warrant further validation in prospective, large-scale, multicenter studies with long-term follow-up. Such research will clarify the long-term efficacy, graft durability, and clinical applicability of both techniques, thereby providing higher-level evidence-based support for the standardized management of complex ureteral strictures.

## Data Availability

The original contributions presented in the study are included in the article/Supplementary Material, further inquiries can be directed to the corresponding author.
